# Demystifying the factors associated with rural–urban gaps in severe acute malnutrition among under-five children in low- and middle-income countries: a decomposition analysis

**DOI:** 10.1038/s41598-020-67570-w

**Published:** 2020-07-07

**Authors:** A. F. Fagbamigbe, N. B. Kandala, A. O. Uthman

**Affiliations:** 1 Department of Epidemiology and Medical Statistics, faculty of Public Health, College of Medicine, University of Ibadan, Ibadan, Nigeria; 2 Division of Health Sciences, Populations, Evidence and Technologies Group, University of Warwick, Coventry, UK; 3 Department of Mathematics, Physics and Electrical Engineering (MPEE), Northumbria University, Newcastle upon Tyne, UK

**Keywords:** Health care, Medical research, Risk factors

## Abstract

What explains the underlying causes of rural–urban differentials in severe acute malnutrition (SAM) among under-five children is poorly exploited, operationalized, studied and understood in low- and middle-income countries (LMIC). We decomposed the rural–urban inequalities in the associated factors of SAM while controlling for individual, household, and neighbourhood factors using datasets from successive demographic and health survey conducted between 2010 and 2018 in 51 LMIC. The data consisted of 532,680 under-five children nested within 55,823 neighbourhoods across the 51 countries. We applied the Blinder–Oaxaca decomposition technique to quantify the contribution of various associated factors to the observed rural–urban disparities in SAM. In all, 69% of the children lived in rural areas, ranging from 16% in Gabon to 81% in Chad. The overall prevalence of SAM among rural children was 4.8% compared with 4.2% among urban children. SAM prevalence in rural areas was highest in Timor-Leste (11.1%) while the highest urban prevalence was in Honduras (8.5%). Nine countries had statistically significant pro-rural (significantly higher odds of SAM in rural areas) inequality while only Tajikistan and Malawi showed statistically significant pro-urban inequality (*p* < 0.05). Overall, neighbourhood socioeconomic status, wealth index, toilet types and sources of drinking water were the most significant contributors to pro-rural inequalities. Other contributors to the pro-rural inequalities are birth weight, maternal age and maternal education. Pro-urban inequalities were mostly affected by neighbourhood socioeconomic status and wealth index. Having SAM among under-five children was explained by the individual-, household- and neighbourhood-level factors. However, we found variations in the contributions of these factors. The rural–urban dichotomy in the prevalence of SAM was generally significant with higher odds found in the rural areas. Our findings suggest the need for urgent intervention on child nutrition in the rural areas of most LMIC.

## Introduction

Childhood malnutrition has remained a major public health challenge in developing countries^1^ and has constituted a long-time barrier to a healthy life and constant threat to human capital development^2^. Severe acute malnutrition (SAM) is one of the worst nutritional outcomes among children worldwide^3,4^. Nutritional outcomes among children have been reported to be influenced by rural–urban differentials in the place of residence of children^5–10^. It is widely noted that children raised in urban areas are generally healthier—in terms of nutritional outcomes than their rural counterparts^2,6^. A substantial body of empirical studies showed that average child’s nutrition outcomes are significantly better in urban areas than in rural areas in most developing countries^1,2,6,11–13^. Studies have suggested that the recent trend of urbanisation across developing countries could have gradually worn off and turn around the rural–urban gaps and create greater nutritional inequalities in urban areas^5^. The recent rapid pace of urbanization in most countries, as well as the occurrence of child malnutrition in urban areas, have not been well explored in low- and middle-income countries (LMIC). The influence of urbanization in child nutrition is two-sided. While higher exposure to the contaminated environment from pollution and wastes in urban areas may increase odds of poor outcomes, access to balanced meals, better housing, health services, safe water and hygiene may put urban children at lower odds of malnutrition^14,15^.

Factors associated with nutritional outcomes in rural and urban areas have been discussed in the literature^5,16–21^. For instance, Srinivasan et al. found differences in the levels of socio-economic characteristics regarding rural and urban children^5^. The authors reported that parental education and the household wealth index contributed a major share of rural–urban disparities in the lowest quantiles of child nutrition outcomes^5^. They affirmed that poor socioeconomic characteristics account for a quarter of rural–urban disparities in the distribution of underweight among under-five children. Sharaf et al. found that the inequalities in child malnutrition between urban and rural areas are explained by differences in the standards of living of the residents in the two different settings^16^. Also, findings elsewhere showed that poor accessibility to media and health care facilities in rural areas put children at higher risk of poorer health outcomes^19–21^.

The rural–urban gaps in nutritional outcomes among children beckon the need for a good understanding of the contributors to the rural–urban gaps in SAM as well as the magnitude of their contributions. The magnitude and the key drivers of these gaps have not been well established across most LMIC. While a couple of studies have carried out the intra-country rural–urban decomposition of nutritional outcomes among children^2,5,13,16–18^, we are not aware of any study that explored the rural–urban inequalities in SAM among multiple countries, especially among the LMIC. Besides, what explains the underlying causes of rural–urban differentials in SAM among under-five children is poorly exploited, operationalized, studied and understood in the LMIC. The extent, pattern and the drivers of the gaps in the rural–urban prevalence of SAM among children in LMIC understanding are not known. We hypothesised that there exist factors that influence these rural–urban gaps in children having SAM. To understand what explains the rural–urban inequalities in the prevalence of SAM among under-five children and make suitable recommendations to provoke discuss and necessary interventions, we assessed and quantified the factors associated to rural–urban inequalities in having SAM among under-five children in 51 LMIC.

The goal of the current study is to establish the magnitude of the gaps in rural–urban inequalities in SAM across the LMIC and identify factors contributing to the gaps to provide an evidence-based answer to critical health policy questions on whether different interventions, policies and approaches are necessary to cub SAM in the rural and urban areas across the low- and middle-income countries. The study also sought to assess the effect of urbanization on the prevalence of SAM among under-five children. Closing the rural–urban inequalities in SAM among under-five children requires a detailed understanding of the main drivers of the inequalities in child nutrition outcomes, especially SAM in LMIC.

## Methods

### Study design and data

We used cross-sectional data obtained from Demographic and Health Surveys (DHS) conducted between 2010 and 2018 and available as of March 2019—when data analysis started—and that included modules on child health. We chose 2010 to focus on recent surveys in the last decade to allow for comparability. The DHS data are nationally representative household surveys conducted in most LMIC. The surveys have similar methodologies and questions in different countries where the surveys held. The DHS uses a multi-stage, stratified cluster sampling design with enumeration areas as the Primary Sampling Units (PSU) with households at the last stage of sampling^22–24^. Due to differences in the political and geographical structures across the countries, there are slight variations in the sampling methodologies across the countries. Country-specific sampling methodologies and reports of findings are available at dhsprogram.com^25–27^. All eligible women and men within each sampled household were interviewed. Sampling weights were calculated based on the population in each stratum to account for unequal selection probabilities whose application makes survey findings to adequately represent the entire population of each country. This is due to the non-proportional allocation of the sample sizes in the different regions and clusters within the same country and the possible differences in response rates. Sampling weights were required for all analysis of the DHS data to ensure the actual representativeness of the survey results at the national levels as well as the sub-national levels. The DHS questionnaires were standardized and implemented across the LMIC with similar interviewer training, supervision, and implementation protocols. The LMIC were determined using the DHS and the World Bank’s categorizations of countries income. For more details, see www.dhsprogram.com; https://data.worldbank.org/income-level/low-and-middle-income and https://datatopics.worldbank.org/world-development-indicators/stories/the-classification-of-countries-by-income.html. The DHS presents the data from each survey in different formats such as women data, child data, birth data, men data, household data etc. In this study, we used the children recode data which was dedicated to health indices of under-five children born to the sampled women within 5 years preceding the survey dates.

### Dependent variable

The severe acute malnutrition is the dependent variable in this study. We defined SAM as “a very low weight-for-height score (WHZ) below −3 z-scores of the median WHO growth standards, by visible severe wasting, or by the presence of nutritional oedema”^4^. It is a composite score of children’ weight and height (weight-for-height). The anthropometry measurements were taken using standard procedures. We generated z-scores by applying the WHO-approved methodologies^28^ to these measurements and categorized children with z-scores < −3 standard deviation as having SAM. SAM is, therefore, a variable with binary outcomes coded as 0 for not having SAM and 1 for those having SAM.

### Main determinant variable

The main determinant variable is the place of residence of the children. The mothers’ place of residence was classified as either rural or urban as of the time of the survey by the DHS using standard procedures with minimal differences in what rural areas were across the countries. For more details, see www.dhsprogram.com.

### Independent variables

The independent variables used in the study were based on the identified factors associated with malnutrition in the literature^2,5,16–18,29,30^. We categorized the factors into individual-level and neighbourhood-level factors.

Individual-level factors include both children, mothers and households characteristics: the sex of the children (male versus female), children age in years (under 1 year and 12–59 months), maternal age (15–24, 25–34, 35–49), occupation (currently working or not), access to media (at least one of radio, television and newspaper), sources of drinking water (improved or unimproved sources), toilet type (improved or unimproved type), weight at birth (average +, small and very small), household wealth index (poorest, poorer, middle, richer and richest), birth interval (firstborn, < 36 months and > 36 months) and birth order (1, 2, 3 and 4 +), recent episode of diarrhoea (yes/no), how soon a child was put to the breast after birth (immediately, within 1 day and after 1 day), availability of health services (whether distances to the health facility was a problem or not), affordability of health services (able to pay for health services or not). However, due to the non-availability of some variables in some countries, the availability and affordability of health services were dropped in the decomposition analysis.

#### Neighbourhood-level factors

The neighbourhood factors were based on the stratum (enumeration areas or geographical clustering) where the children lived. Neighbourhoods were based on sharing a common PSU (enumeration area) within the DHS sampling frame^22,23^. Operationally, we defined “neighbourhood” as clusters and “neighbours” as members of the same cluster. The PSUs were identified using the most recent census in each country. We computed the neighbourhood socioeconomic disadvantage composite score using principal component analysis of the proportion of respondents within each neighbourhood who are illiterates, poor, and unemployed.

### Statistical analyses

**Figure 1:**
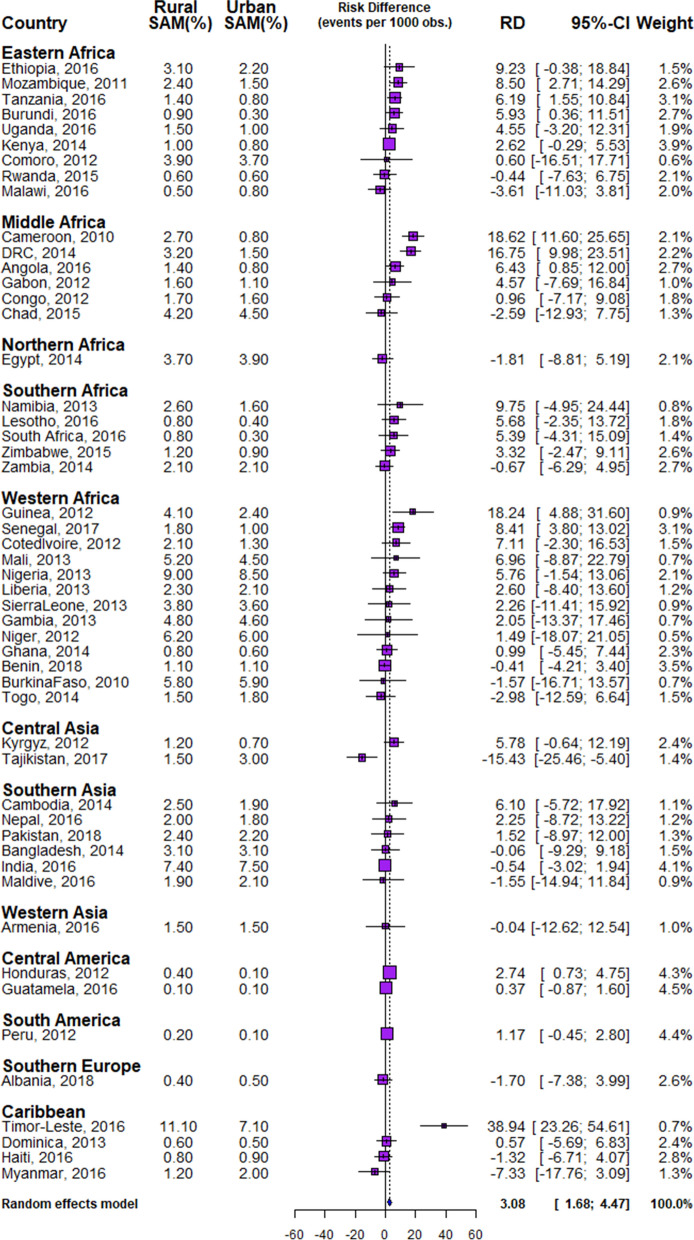
Forest plot of the risk difference in the prevalence of SAM between rural and urban children by countries.

**Figure 2:**
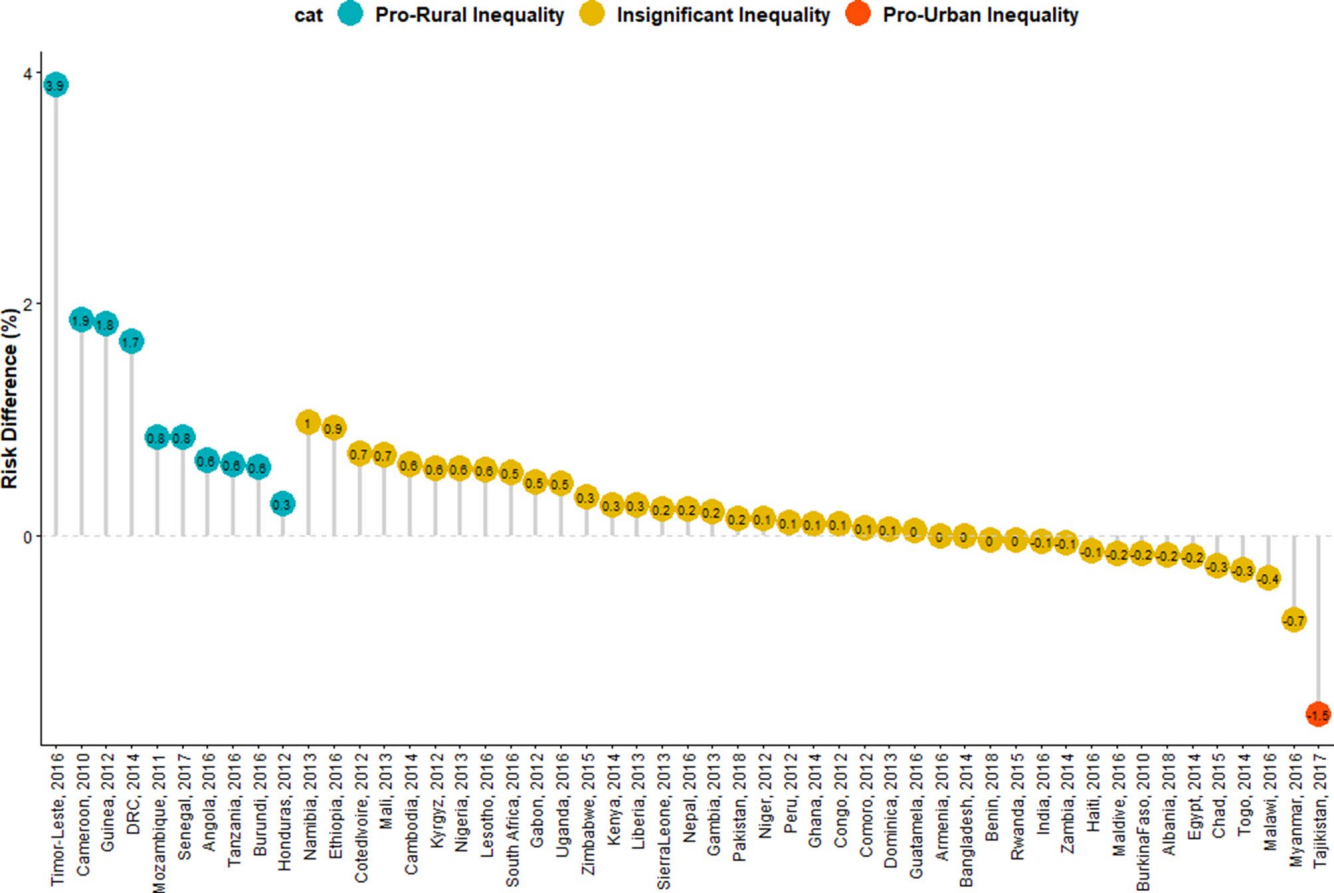
Risk difference between children born to rural and urban mothers in the prevalence of SAM by countries.

**Figure 3:**
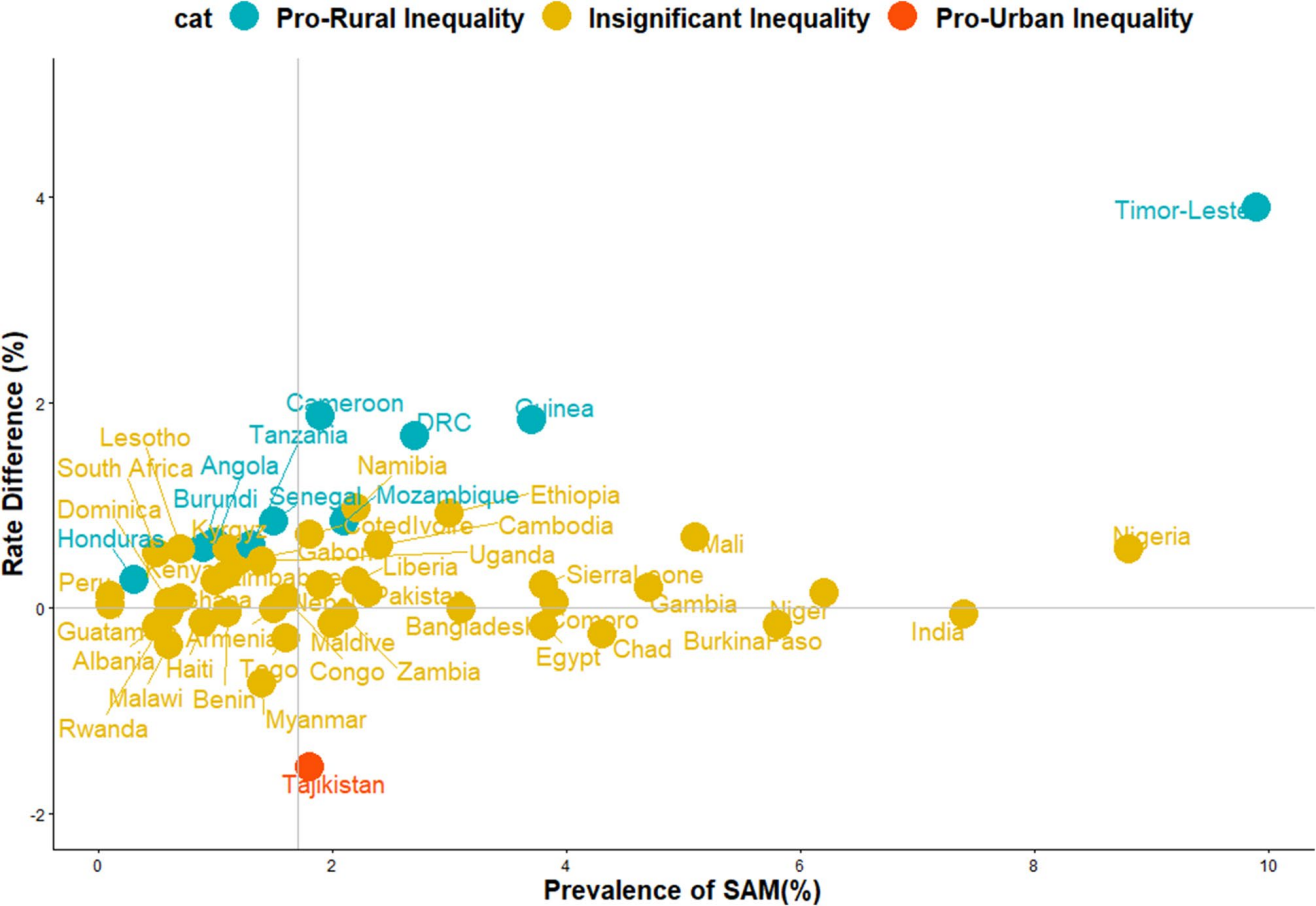
Scatter plot of rate of SAM and risk difference between children born to rural and urban mothers in LMIC.

**Figure 4:**
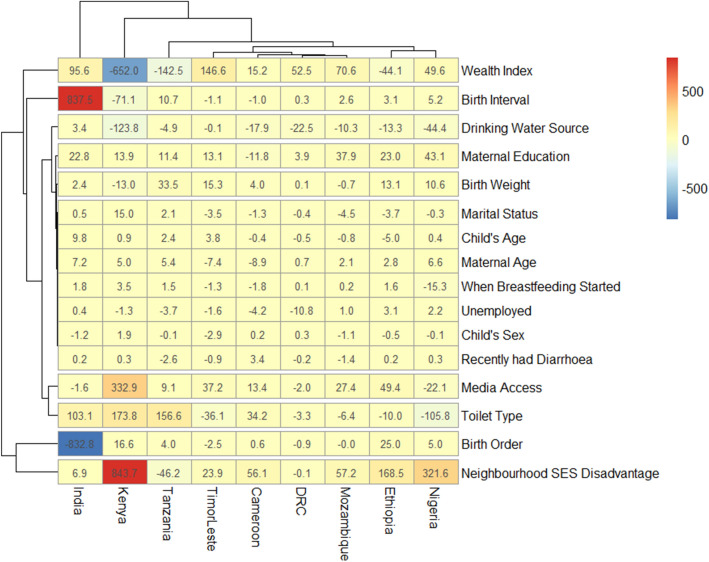
Contributions of differences in the distribution ‘compositional effect’ of the determinants of SAM to the total gap between children from rural and urban mothers by the pro-rural inequality countries.

**Figure 5:**
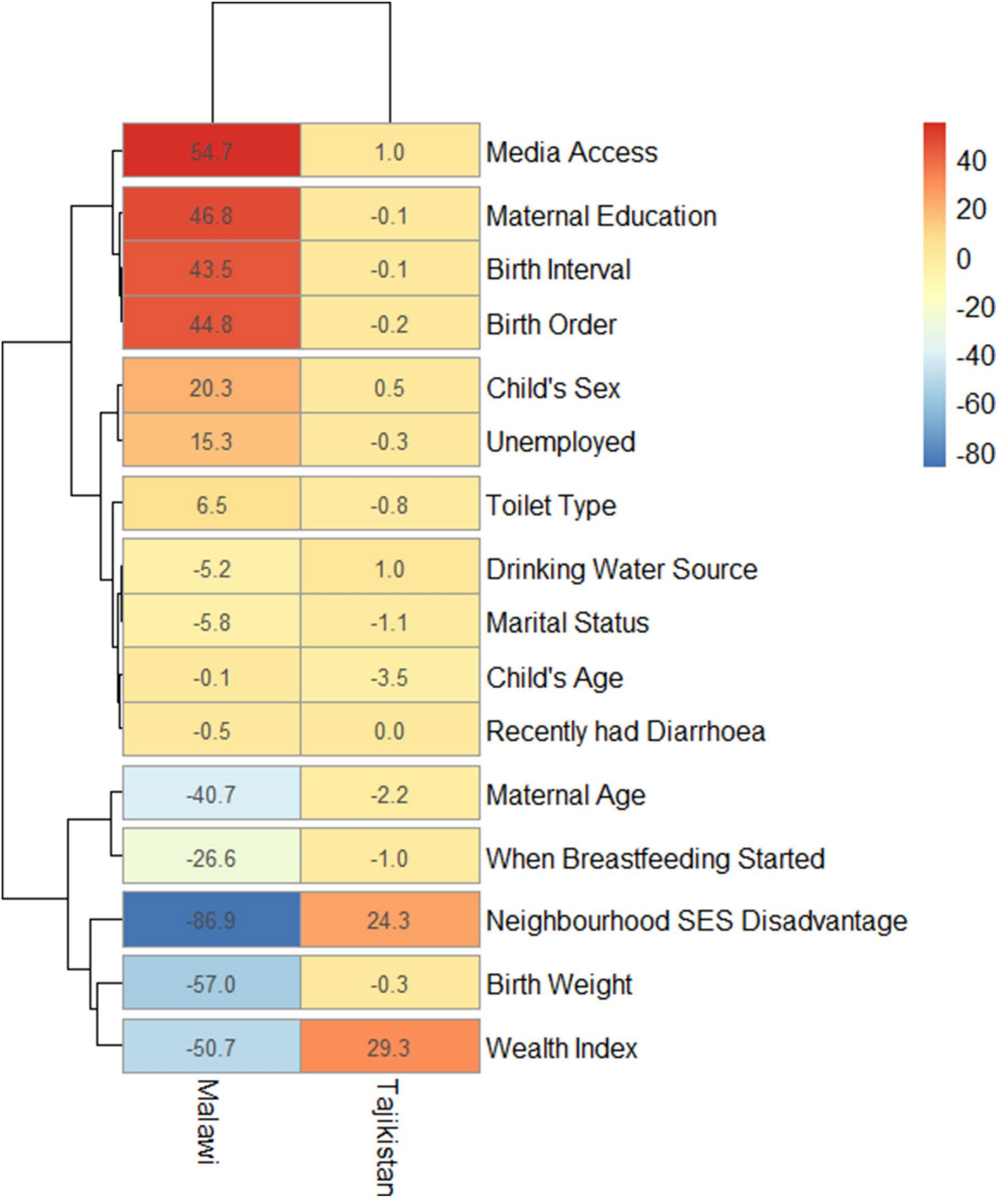
Contributions of differences in the distribution of ‘compositional effect’ of the determinants of SAM to the total gap between children from rural and urban areas by the pro-urban inequality countries.

In all, data of 532,680 under-five children nested within 55,823 neighbourhoods from 51 LMIC who participated in the DHSs between 2010 and 2018 were analysed. We carried out analytical analyses comprising of univariable analysis, bivariable analysis and multivariate analysis for Blinder–Oaxaca decomposition techniques with binary multivariable logistic regression model. Univariable and bivariable analysis were used to show the distribution of respondents by their countries, the distribution of SAM and the independent variables. We computed the risk difference (RD) in the prevalence of SAM between rural and urban under-five children. Any RD greater than 0 suggests that SAM are more prevalent among children in rural areas (pro-rural inequality). Conversely, a negative RD indicates that SAM is prevalent among children in urban areas (pro-urban inequality). All the descriptive statistics: distribution of characteristics, prevalence and RD were weighted. We computed the random effect of RD in SAM among rural and urban children (Fig. 1). The random effect shows the overall risk difference among all children irrespective of their countries. In Figs. 2 and 3, we displayed the distribution of RD by countries using colours blue, orange and red to indicate statistically significant pro-rural inequality, no significant inequality and statistically significant pro-urban inequality respectively. Finally, the binary multivariable logistic regression model using the pooled cross-sectional data of SAM from the 51 LMIC was used to carry out a Blinder–Oaxaca decomposition analysis of rural and urban differentials in SAM. Figures 4 and 5 shows the decomposition analysis for pro-rural and pro-urban countries rspectively.

### Blinder–Oaxaca decomposition analysis

The Blinder–Oaxaca decomposition is a statistical analysis methodology with an assumption that children born to rural mothers had the same characteristics as children born to urban women^31,32^. The method allows for the decomposition of the differences in an outcome variable between 2 groups into 2 components so that the gaps can be seen and understood more clearly. It identifies two sources of outcome differentials between groups^19,31,33–36^. The first component of the decomposition is the “explained” portion of the gap that captures differences in the distributions of the measurable characteristics (also known as the “compositional” or “endowments”) of these groups. This method enabled the quantification of how much of the gap between the “advantaged” and the “disadvantaged” groups is attributable to differences in specific measurable characteristics. The second component is the “unexplained” part (also referred to as the structural component or return effect) which captured the gap due to the differences in the regression coefficients and the unmeasured variables between the two groups been compared. This second component is attributed to differences in the returns to endowments between groups. So each group had different returns for the same level of endowments^19,31,33–36^. It was initially built for continuous outcomes but has been extended to analyse non-linear outcomes including binary outcomes which are the most prevalent forms of outcomes in health outcomes and behaviours. For instance, Asuman et al. extended the technique to decompose differentials between rural and urban children who took or did not take immunisation^33^. In the current study, the non-linear decomposition model assumes that the conditional expectation of the probability of a child having SAM is a non-linear function of a vector of characteristics. The results of the decomposition analysis were presented in Figs. 4 and 5. The “explained” (compositional component) and the “unexplained” (structural component) portions of the educational inequalities are depicted by red and blue colours respectively; the lighter the red colour, the lower the percentage contribution of the “explained” portion and the lighter the blue colour, the lower the percentage contribution of the “unexplained” portion. All statistical analyses were carried out using Stata 16 and R statistical software.

The study flowchart is summarized thus (1) Pool all the data that meet the inclusion criteria (2) determine the prevalence of SAM by rural and urban areas in each country (3) determine the prevalence of SAM by rural and urban areas by the children demographics (4) find the risk differences (the differences in prevalence between rural areas and urban areas in each country) in SAM and display the risk differences for each country to ensure good understanding by the readers (5) determine the pro-rural countries (countries with significantly higher prevalence in rural areas) and the pro-urban countries (countries with significantly higher prevalence in urban areas) and (6) decompose factors associated with rural–urban inequalities in SAM.

Ethical approvals were obtained from the Ethics Committee of the ICF Macro at Fairfax, Virginia in the USA and by the National Ethics Committees in the participating countries. Written and signed informed consent was obtained from each parent and/or legal guardians of the children who participated in the study were told that the interviews have minimal risks and potential benefits. All information was collected anonymously and held confidentially. The full ethical approval details have been reported earlier^25,27,37^ and can be found at https://dhsprogram.com.

### Ethics approval and consent to participate

This study was based on an analysis of existing survey data with all identifier information removed. The surveys were approved by the Ethics Committee of the ICF Macro at Fairfax, Virginia in the USA and by the National Ethics Committees in the participating countries. The full details can be found at https://dhsprogram.com. All methods for data collection and data analysis were carried out following relevant guidelines and regulations on the protection of participants’ data.

## Results

### Sample characteristics

The names of the countries, year of data collection, numbers of neighbourhoods, number of under-five children and the weighted prevalence of SAM and percentage of mothers from rural areas are listed in Table 1. The overall proportion of children from rural areas was 69.3% and ranged from 16% in Gabon to 81% in Chad.

**Table 1:** Description of Demographic and Health Surveys data by countries and SAM prevalence among under-five children in LMIC by rural–urban residence, 2010–2018.

Country	Year of survey	Number of under-5 children	Weighted rural (%)	Weighted SAM prevalence (%)	SAM (%) rural	SAM (%) urban
All		532,680	69.3	4.7	4.8	4.2*
**Eastern Africa**		67,418	77.4	1.5	1.7	1.1
Burundi	2016	6,052	91.1	0.9	1.0	0.3
Comoro	2012	2,387	73.4	3.9	3.9	3.7
Ethiopia	2016	8,919	89.1	3.0	3.1	2.2*
Kenya	2014	18,656	65.7	1.0	1.0	0.8*
Malawi	2016	5,178	87.1	0.6	0.5	0.8
Mozambique	2011	9,313	73.1	2.1	2.4	1.5*
Rwanda	2015	3,538	83.6	0.6	0.6	0.6
Tanzania	2016	8,962	74.2	1.3	1.4	0.8*
Uganda	2016	4,413	79.7	1.4	1.5	1.0
**Middle Africa**		37,136	58.5	2.5	3.2	1.6
Angola	2016	6,407	40.0	1.0	1.4	0.8
Cameroon	2010	5,033	56.7	1.9	2.7	0.8
Chad	2015	9,826	80.8	4.3	4.2	4.5
Congo	2012	4,475	40.5	1.6	1.7	1.6
DRC	2014	8,059	69.5	2.7	3.2	1.5*
Gabon	2012	3,336	16.0	1.2	1.6	1.1
**Northern Africa**		13,682	69.2	3.8	3.7	3.9
Egypt	2014	13,682	69.2	3.8	3.7	3.9
**Southern Africa**		20,273	66.2	1.7	1.7	1.6
Lesotho	2016	1,312	72.6	0.7	0.8	0.4
Namibia	2013	1,558	56.6	2.2	2.6	1.6
South Africa	2016	1,082	43.4	0.5	0.8	0.3
Zambia	2014	11,407	66.8	2.1	2.1	2.1
Zimbabwe	2015	4,914	70.4	1.1	1.2	0.9
**Western Africa**		85,462	67.1	4.7	4.9	4.3
Benin	2018	12,033	61.2	1.1	1.1	1.1
Burkina Faso	2010	6,532	83.1	5.8	5.8	5.9
Cote d’Ivoire	2012	3,200	64.4	1.8	2.1	1.4
Gambia	2013	3,098	55.8	4.7	4.8	4.6
Ghana	2014	2,720	54.5	0.7	0.8	0.6
Guinea	2012	3,085	75.1	3.7	4.1	2.4
Liberia	2013	3,171	49.5	2.2	2.3	2.1
Mali	2013	4,306	80.7	5.1	5.2	4.5
Niger	2012	4,771	87.1	6.2	6.2	6.0
Nigeria	2013	24,505	63.0	8.8	9.1	8.5*
Senegal	2017	10,787	63.8	1.5	1.8	1.0
Sierra Leone	2013	4,069	77.3	3.8	3.8	3.6
Togo	2014	3,185	65.4	1.6	1.5	1.8
**Central Asia**		9,883	76.4	1.5	1.4	2.0
Kyrgyz	2012	4,016	71.5	1.1	1.3	0.7
Tajikistan	2017	5,867	79.3	1.8	1.5	3.0
**Southern Asia**		245,173	72.2	2.4	7.1	7.1
Cambodia	2014	4,324	85.7	2.4	2.5	1.9
Bangladesh	2014	6,965	74.8	3.1	3.1	3.1
India	2016	225,002	72.2	7.4	7.4	7.5*
Maldives	2016	2,362	69.2	2.0	1.9	2.1
Nepal	2016	2,369	47.0	1.9	2.0	1.8
Pakistan	2018	4,151	67.3	2.3	2.4	2.2
**Western Asia**		1561	43.6	1.5	1.5	1.5
Armenia	2016	1561	43.6	1.5	1.5	1.5
**Central America**		21,717	60.2	0.2	0.2	0.1
Guatemala	2012	11,744	64.3	0.1	0.1	0.1
Honduras	2016	9,973	54.8	0.3	0.4	0.1
**South America**		9,213	34.6	0.1	0.2	0.1
Peru	2012	9,213	34.6	0.1	0.2	0.1
**South Europe**		2,462	45.0	0.5	0.4	0.5
Albania	2018	2,462	45.0	0.5	0.4	0.5
**Caribbean**		18,700	63.4	3.9	4.6	2.5
Dominica	2013	3,187	25.9	0.6	0.6	0.6
Haiti	2016	5,598	66.5	0.9	0.8	0.9
Myanmar	2016	4,197	78.1	1.4	1.2	2.0
Timor-Leste	2016	5,718	71.3	9.9	11.1	7.1*

*Significant at 0.05 in Mantel Haenszel test of homogeneity of the odds ratio.

### Prevalence of SAM

The prevalence of SAM among rural and urban children across the 51 LMIC studied are shown in Table 1 and Fig. 1. The overall SAM prevalence was 4.7% with a median prevalence of 1.8%, it ranged from 0.1% in Guatemala to 9.9% in Timor-Leste as shown in Table 1. The prevalence of SAM among children of mothers residing in rural areas was 4.8%, ranged from 0.1% in Guatemala to 11.1% in Timor-Leste, while the overall prevalence in the urban areas was 4.2%, ranging from 0.1% in Peru, Guatemala and Honduras to 8.5% in Nigeria.

The descriptive statistics for the pooled sample of children across the 51 LMIC by selected characteristics are shown in Table 2. About 49% of the children were females while only 20% were infants. About 31% and 53% of the mothers were aged between 15–24 years and 25–34 years respectively while about 31% had no formal education. Also, the prevalence of SAM among infants from rural areas was 7.7% compared with 6.7% from urban areas. Mantel Haenszel test of homogeneity of odds ratio using the rural–urban prevalence of SAM as an effect modifier showed that all characteristics considered were independently significant (*p* < 0.05).

**Table 2:** Summary of pooled sample characteristics of the studied children in 51 LMIC by rural–urban residence.

Characteristics	Weighted n	Weighted %	Weighted (%) rural (%)	SAM (%) rural	SAM (%) urban
Individual level	532,680	100	69.3	4.8	4.2*
**Age**					
< 12 months	103,379	20.0	69.8	7.7	6.7*
12–59 months	413,718	80.0	69.1	4.2	3.6
**Sex**					
Female	252,541	48.8	69.4	4.5	3.9*
Male	264,556	51.2	69.4	5.3	4.5
**Maternal age**					
15–24	160,133	31.0	72.0	5.5	4.5*
25–34	273,802	52.9	67.4	4.8	4.3
35–49	83,162	16.1	70.1	4.0	3.4
**Maternal education**					
None	165,629	31.1	83.8	5.9	5.6*
Primary	134,578	25.3	74.0	3.1	2.9
Secondary +	231,738	43.6	56.1	5.2	4.3
**Wealth Index**					
Poorest	122,991	23.8	93.7	5.7	4.6*
Poorer	112,755	21.8	87.7	4.8	4.8
Middle	104,194	20.1	74.7	4.5	4.4
Richer	96,896	18.6	50.2	4.0	4.5
Richest	80,261	15.5	22.0	3.9	3.8
**Employment**					
Yes	366,033	70.8	70.1	5.2	4.5*
No	151,064	29.2	67.2	4.1	3.5
**Access to media**					
No	188,357	36.5	86.1	5.4	4.4*
Yes	328,311	63.5	89.6	4.4	4.2
**Drinking-water sources**					
Unimproved	95,544	19.2	86.9	4.3	3.0*
Improved	402,688	80.8	65.0	5.0	4.3
**Toilet type**					
Unimproved	248,331	49.9	85.9	5.3	4.2*
Improved	249,753	50.1	52.6	4.0	4.2
**Marital status**					
Never married	12,199	2.3	52.5	2.1	1.6*
Currently married	484,949	93.8	70.0	5.0	4.4
Formerly married	19,946	3.9	60.9	2.8	1.8
**Weight at birth**					
Average +	423,017	85.4	68.6	4.8	4.3*
Small	52,939	10.6	71.3	5.4	4.0
Very small	19,624	4.0	72.9	6.6	5.7
**Birth interval**					
1st	157,067	30.4	64.6	5.1	4.3*
< 36	193,030	37.4	74.6	5.0	4.7
36 +	165,780	32.2	67.5	4.5	3.6
**Birth order**					
1	157,065	30.4	64.6	5.1	4.3*
2	134,436	26.0	66.2	5.2	4.3
3	83,134	16.1	69.7	4.9	4.1
4	142,462	27.5	77.1	4.5	4.0
**Breastfeeding started**					
Immediately	236,717	47.1	69.8	4.6	4.4*
First day	200,539	39.9	69.1	5.2	4.2
After 1st day	65,848	13.0	70.0	5.4	4.1
**Distance to facility**					
No problem	86,173	17.3	55.7	6.1	5.9*
Problem	411,221	82.7	71.9	4.8	3.8
**Had diarrhoea recently**					
No	463,975	87.3	69.2	4.9	4.3*
Yes	67,197	12.7	69.8	5.0	3.6
**Can afford healthcare**					
Yes	101,954	20.5	63.2	6.5	6.2*
No	395,445	79.5	70.6	4.7	3.8
**Year**					
2010	12,050	2.3	71.7	4.7	2.5*
2011	10,179	1.9	73.1	2.4	1.5
2012	43,014	8.1	55.6	2.5	0.9
2013	44,495	8.4	62.0	6.9	5.8
2014	69,379	13.0	68.9	2.4	2.0
2015	19,099	3.6	78.5	2.7	2.5
2016	298,787	56.2	72.1	6.0	5.9
2017	16,650	3.1	69.7	1.7	1.5
2018	18,319	3.4	60.3	1.3	1.2
**Community SES status**					
1 (highest)	120,219	22.6	35.7	4.5	3.9*
2	103,925	19.5	58.2	3.9	3.7
3	105,628	19.9	74.1	4.1	5.2
4	103,069	19.4	88.9	4.9	5.6
5 (lowest)	99,131	18.6	95.9	6.4	5.8
Total	532,680	100.0	69.3	4.8	4.2*

*Significant at 0.05 in Mantel Haenszel test of homogeneity of the odds ratio.

### Magnitude and variations in rural–urban inequality in SAM

We present the differences, a measure of inequality, in the risk of having SAM among children from rural and urban areas across the countries in Figs. 1 and 2. The prevalence of SAM was higher in the rural areas than in the urban areas in all the countries except in Malawi, Rwanda, Chad, Egypt, Zambia, Benin, Burkina Faso, Togo, Tajikistan, Bangladesh, India, Maldives, Armenia, Albania, Haiti and Myanmar. In Eastern Africa, the pro-rural risk difference (RD) in SAM were largest in Ethiopia (9.23 per 1,000 children) and pro-urban was highest for Malawi (− 3.61). In Western Africa, the largest pro-rural difference was in Guinea (18.24) and pro-urban was highest for Togo (− 2.98). In the Caribbean, the pro-rural risk difference was largest for Timor Leste (38.94) and the pro-urban risk difference was highest for Myanmar (− 7.33). India (4.1%), Honduras (4.3%), Guatemala (4.5%), and Peru (4.4%) had the greatest weight contribution to the pooled random effect. In the pooled analysis, Timor Leste with a risk difference of 38.94 per 1,000 children, Guinea (18.24), Cameroon (18.62) and DRC with 16.75 had the highest pro-rural inequalities compared with highest pro-urban inequality in Tajikistan (− 15.43). Overall, there was significant pro-rural inequality among all the sampled children, with a risk difference of 3.08 (95% CI 1.68–4.47) per 1,000 children as shown in the random effects in Fig. 1.

Based on risk differences, three of the nine countries in Eastern Africa, 2 of the countries in Middle Africa, none in Northern Africa and Southern Africa showed statistically significant pro-rural inequality. Of the 13 countries in Western Africa, only Guinea and Senegal showed statistically significant pro-rural inequality while no country in Central Asia, Southern Asia, Western Asia, and in Southern Europe had pro-rural inequality. Honduras in Central America and Timor-Leste in the Caribbean showed statistically significant (*p* < 0.001) pro-rural inequality. Tajikistan is the only country that has statistically significant (*p* < 0.001) pro-urban inequality in the prevalence of SAM (Figs. 1, 2, 3).

### Relationship between prevalence of SAM and magnitude of inequality

Figure 3 shows the relationship between the prevalence of SAM and the magnitude of rural–urban inequality for all the 51 countries in this study. We categorized the countries into 4 distinct categories based on this relationship.

1. High SAM and high pro-rural inequality such as Cameroon, DRC, Timor-Leste and Guinea;
2. High SAM and high pro-urban inequality such as in Tajikistan;
3. Low SAM and high pro-rural inequality such as Senegal, Burundi, and Angola;
4. Low SAM and high pro-urban inequality such as Myanmar.

### Decomposition of rural–urban inequality in the prevalence of SAM

Among the 51 LMIC included in this decomposition analysis, 9 countries (India, Kenya, Tanzania, Timor-Leste, Cameroon, Democratic Republic of Congo, Mozambique, Ethiopia and Nigeria) showed statistically significant pro-rural inequality while only Tajikistan and Malawi showed statistically significant pro-urban inequality as shown in Figs. 4 and 5 respectively. It is worth noting that while Malawi had insignificant pro-urban inequality when RD was used (Fig. 3), it was significant in the decomposition analysis using odds ratios (Fig. 5). The Figs. 4 and 5 show the detailed decomposition of the part of the rural–urban inequality that was caused by compositional effects of the determinants of SAM among under-five children. There were variations in the factors associated with the pro-rural across the nine pro-rural countries. Generally, neighbourhood socioeconomic status disadvantage, birth order, birth interval, household wealth index, sources of drinking water as well as mothers’ educational attainment, access to media and the type of toilet in the households had highest contributions to the rural–urban gap in SAM in most countries.

For instance, birth order and birth interval were the largest contributors to rural–urban differentials in children having SAM in India. Put together, the two factors contributed over 90% of the inequality in that country (Fig. 4). Other lesser contributors are toilet type and household wealth index. In Kenya, the largest contributions to pro-rural inequality in the prevalence of SAM were made by neighbourhood socioeconomic disadvantage, followed by wealth index, access to media, toilet type and source of drinking water. In Nigeria, the greatest contributors to the rural–urban disparities were mainly neighbourhood socioeconomic disadvantage and toilet type. Other smaller contributors are wealth index, mother educational attainment, drinking water sources and access to media. In Timor-Leste, wealth index had the largest contribution to the rural–urban inequality followed by toilet type and neighbourhood socioeconomic status disadvantage whereas toilet type held sway in Tanzania followed by wealth index and neighbourhood socioeconomic status disadvantage. Other factors such as birth weight, children age and sex, maternal employment status, marital status, age and educational attainment had the lowest contribution to rural–urban inequality in the prevalence of SAM across these countries.

For the countries with pro-urban inequality, the household wealth index and neighbourhood socioeconomic disadvantage status contributed mainly to why the odds of SAM was higher among children in urban areas than among those in the rural areas (Fig. 5). Other contributors to the inequality include media access, maternal education and age, birth weight, birth interval and birth order. In Malawi, pro-urban inequalities were mainly explained by media access, mothers’ educational attainment, birth interval, birth order, neighbourhood socioeconomic status disadvantage, childbirth weight and household wealth index. Only neighbourhood socioeconomic status disadvantage and household wealth index were the dominant contributors to a higher risk of SAM among urban children than the rural children in Tajikistan.

## Discussions

In this study, we found wide variations in rural–urban inequality in the distribution of severe acute malnutrition among under-five children from the 51 low- and middle-income countries. Also, we quantified the contribution of various associated factors to the rural–urban gaps in SAM in LMICs. Similar to previous reports, we found a wide range of factors that were associated with the prevalence of SAM in LMIC^5,17,18^. These factors also contributed to rural–urban differences in SAM among under-five children in LMICs. Neighbourhood socioeconomic status disadvantage, birth order, birth interval, household wealth index and sources of drinking water were the largest contributors to pro-rural inequalities, while neighbourhood socioeconomic status disadvantage and household wealth index were the major contributors to pro-urban inequality. Our finding agrees with reports of Mussa et al. which found significant differences in the determinants of child malnutrition among under-five children between urban and rural areas in Malawi^17^ and Novengnon et al. which focussed on the decomposition of inequalities in child malnutrition urban and rural areas^18^.

We found significant inter-country variations in the risk-difference in the prevalence of SAM among children in both urban and rural areas. In most of the countries, the prevalence of SAM was higher in the rural areas, except Malawi, Rwanda, Chad, Egypt, Zambia, Benin, Burkina Faso, Togo, Tajikistan, Bangladesh, India, Maldives, Armenia, Albania, Haiti and Myanmar where SAM prevalence was higher in urban areas. Overall, we found pro-rural inequality in SAM among the children, irrespective of their countries and regions with a pro-rural risk difference of 3 per 1,000 children. Considering the regions, the greatest pro-rural inequality was observed in the Caribbean (Timor-Leste) while the greatest pro-urban inequality was observed in Central Asia (Tajikistan). The magnitudes of these inequalities are alarming and suggest neglect in health issues of children in rural areas compared with their urban counterparts. Studies have suggested that people who live in urban areas are at higher advantage of better medical care due in part to better awareness, proximity to health care facilities and better access to health practitioners^2,21,38^. Our findings show the need for urgent health interventions that are particularly focused on rural children.

Compared with the urban areas, rural areas may have a unique set of characteristics which put the nutrition of rural children at a disadvantage. We found that children nutritional outcomes in rural areas are affected by the level of parental income, less hygienic environment, large family size, and short birth intervals. Whereas, the urban children are at higher odds of a balanced meal, improved housing schemes, healthcare services, portable water and higher availability of employment an higher pay thereof^2,17^.

Our results revealed unequal distribution in the prevalence of SAM among rural and urban children. This is an indication of inequalities attributable to the location of residence. Although SAM prevalence was higher in rural areas than urban areas in 35 countries, SAM was significantly prevalent among rural children in 9 countries (pro-rural inequality). Among these countries, the risk difference ranged from 2 per 1,000 children to 16 per 1,000 children. Pro-urban inequality in SAM, although apparent in 16 countries, was significant only in Tajikistan and Malawi. These could be as a result of the adverse effect of urbanization. One would have expected children in urban areas to have better nutritional outcomes but the reverse was the case in these two countries. This finding is corroborated by earlier reports that urban children, despite having better-off conditions, may suffer malnutrition more than rural children as a result of urbanization^5,39,40^. That is, in some cases, media access, education, better-off economic conditions may not be sufficient to avert SAM, the mothers’ nutritional practices and hygiene, sanitation and environmental factors play a crucial role in malnutrition among children. A Nigerian study established that malnutrition affects the urban-poor children disproportionately^40^. This suggests that there may be a need to carry out further evaluation of this finding as slum children in urban areas, whose living conditions may be worse off than those in rural areas, are classified as urban children. Nonetheless, we recommend that urban children should not be left out in aggressive intervention to halt malnutrition among under-five children in LMIC.

The wide pro-urban inequality in Tajikistan is very distinct and interesting. It thus requires further investigation to explore what drove the pro-urban inequality in the country. Our finding aligns with previous studies which reported inequalities in nutritional outcomes among rural children and urban children^2,17,18^. Countries that are experiencing significant rural–urban inequalities may need to explore and take a cue from other countries such as Chad, Haiti, Guatemala and Benin, with insignificant rural–urban inequalities, and take urgent steps to ensure that necessary interventions that could lead to a paradigm shift in under-five nutrition are made to meet the fast-approaching deadline for the targets of the sustainable development goals on health and equality^41^.

Several factors explained the rural–urban gaps in the prevalence of SAM among under-five children. The foremost among these factors is the neighbourhood socioeconomic status disadvantage which generally accounted for higher risk of SAM in rural residence compared to the urban residence in pro-rural countries and vice versa in pro-urban countries. That is, neighbourhood socioeconomic status disadvantage contributed to both the pro-rural and pro-urban inequalities. This finding could be attributed to the fact that mothers of children in urban area have a higher likelihood of financial wherewithal, access to better resources and health information as well as health care services; hence more likely to provide their children with better nutritional foods. Poorer neighbourhoods increase pro-rural inequality while the better-off ones widen the pro-urban inequalities in the likelihood of a child developing SAM.

Notably, children from socioeconomically disadvantaged neighbourhoods, who had drinking water from unimproved sources as well as from mothers with no education, poor access to media and households with unimproved types of toilet, from households in poor wealth quintiles, short birth interval, and higher birth orders were at a higher likelihood of having pro-rural inequalities in developing SAM. On the contrary, children from richest households, from educated mothers, maternal access to any of television, newspaper and magazine significantly reduces the chances of developing SAM and contributed to rural–urban gaps in the development of SAM across the countries. Our findings are consistent with previous reports^6,8–10^. Our finding is quite intuitive as mothers from households in the higher wealth quintiles and educated mothers are better positioned to have access to information that could enable their chances of making good decisions on providing adequate and sufficient nutritious foods to their children. Besides, the higher propensity of the better-off mothers to use health care services and listen to media could reduce the risk of SAM among their children. Also, children with low birth weight were at higher odds of developing SAM in Tanzania. This is a pointer that efforts should be made to avert, or at least reduce low-birthweight as such outcomes have a far-reaching effect on the future health outcomes of the children.

However, how soon a child was put to the breast after birth and whether a child had diarrhoea with 2 weeks preceding the survey did not make any significant contribution to the explanation of neither pro-rural nor pro-urban inequalities. This finding is at variance with existing literature that diarrhoea is a risk factor of malnutrition and that breastfeeding could help prevent malnutrition among children^29,30^. Similarly, the availability and affordability of health services were insignificant. We had used these variables as proxies for nutrition supply/intake, infections and other morbidities as well as the accessibility of health services. We count our limited choice of independent variables as a study limitation because different countries have different restrictions on the type of variables collected.

Our findings have several public health implications for child nutrition and maternal and child healthcare programming in LMIC. Firstly, there is a need for most LMIC to develop all-evolving child nutrition interventions and programmes targeted at rural mothers and their children. Secondly, awareness campaign and health education and promotion on child nutrition are much needed to ease off the inequalities in severe acute malnutrition suffered in rural areas. This has become necessary as a result of poor access to media such as television, radio and newspaper in rural areas. Health education and promotion could be in the form of one-on-one meetings with mothers, radio communication, and town hall meetings and seminars. Political will and adequate involvement of the community and religious leaders could also be helpful. However, urban children should not be left out of such interventions, especially in countries with pro-urban inequality, as we found a high burden of SAM in urban areas at 4.2%, although lower than the 4.8% in the rural areas.

Thirdly, there is a need to evaluate and reinvigorate maternal and child health across these countries, widen the scope of existing policies, consider and involve the different factors identified in the current study in future child nutrition policies. It will be very helpful to engage the identified connection among the structure, composition and the context in which the children live. Our findings underscore the advantage of enhancing both the compositional and structural characteristics that affect the prevalence of SAM among children if the rural–urban inequalities in SAM are to be narrowed.

Another important lesson learnt from this study is that the socio-economic status of the mother and their households explains the differences in SAM between urban and rural areas. Individual-, community- and perhaps country-specific factors such as access to media, household wealth status, country-level policies and programmes for child nutrition, famine, war, internal displacement, political, and economic instability could help explain why some countries such as Senegal, Angola, Burundi had a low SAM prevalence and high pro-rural inequality while countries such as Cameroun, Democratic Republic of Congo, Timor-Leste and Nigeria had high SAM prevalence and high pro-rural inequality. The effect of neighbourhood socioeconomic status on the likelihood of SAM among children is very striking and significant across all the countries. Our finding is corroborated by previous studies that identified children in high socioeconomic areas to have a higher likelihood of better health outcomes^2,11^.

## Study limitations and strengths

Due to the secondary nature of the data used in the current study, we are limited in the choice of our explanatory variables. Also, the data might have suffered recall bias. Our study has considered the rural and urban areas as homogeneous but this may not be the case everywhere as availability of health services as well as health providers and improved water sources vary from communities to communities. More so, there are some slums within urban areas such as the various slums in Nairobi, Kenya. It is not unlikely that the slum children in urban areas, whose living conditions may be worse than those in rural areas, are classified as urban children. The same could be said of pockets of affluent households in rural areas. This may help explain the situation in Malawi and Tajikistan. While the Blinder–Oaxaca decomposition analysis method used in the current study does not infer causality, it nonetheless provides robust evidence of inequalities between the two groups of children after controlling for the exposure variable. The strength of our study are numerous, we have used nationally representative data known for accuracy and comparability in 51 countries, a good method for decomposing factors were used, we were able to quantify the magnitude of the explained and unexplained variations in factors associated with SAM.

## Conclusions

SAM was prevalent in most of the countries. The rural–urban gaps in the prevalence of SAM among under-five children were explained by the individual, household and community-level factors. The rural–urban dichotomy in the prevalence of SAM was generally significant in nine countries having pro-rural inequality and two countries having pro-urban inequality. The overall significance of pro-rural gaps among all the studied children further strengthened our arguments that urgent intervention is essential in rural areas of most LMIC. We strongly agree with previous recommendations in the literature that children in most developing countries should be given health and social protection scheme to ensure equality and equity in child health and eliminate rural–urban discrepancies in childhood health outcomes. Health programmers and policymakers in the LMIC, especially in the countries with pro-rural inequalities must reformulate policies and interventions on child nutrition with a greater focus on rural children as they are the most disadvantaged and vulnerable.

### Informed consent and confidentiality

Written and signed informed consent was obtained from each parent and/or legal guardians of the children who participated in the study were told that the interviews have minimal risks and potential benefits and that information will be collected anonymously and held confidentially. The full details can be found at https://dhsprogram.com.
